# Developing a framework for performance assessment of the public long-term care system in Korea: methodological and policy lessons

**DOI:** 10.1186/s12961-020-0529-8

**Published:** 2020-02-22

**Authors:** Hongsoo Kim, Boyoung Jeon

**Affiliations:** 1 Graduate School of Public Health Dept. of Public Health Sciences, Institute of Aging, and Institute of Health and Environment, Seoul National University, Seoul, 151-742 Republic of Korea; 2 National Rehabilitation Center, Seoul, 01022 Republic of Korea

**Keywords:** Long-term care systems, performance measurement, social long-term care insurance

## Abstract

**Background:**

Limited evidence exists on how to assess long-term care system performance. This study aims to report on the process and results of developing a performance assessment framework to evaluate the long-term care system financed by the public long-term care insurance in South Korea.

**Methods:**

The framework was developed through a six-step approach, including setting the goals and scope of performance assessment in the given policy context, reviewing existing performance frameworks, developing a framework with a wide range of potential indicators, refining the framework through a series of Delphi surveys and expert meetings, examining the feasibility of generated indicators through a pilot test, receiving the comments of stakeholders, and finalising the performance framework.

**Results:**

The finalised framework has 4 domains – coverage, quality of care, quality of life and system sustainability – and 28 indicators, including 10 core indicators to monitor long-term care system performance. Usability and feasibility along with policy relevance were important criteria in selecting these indicators. The proposed framework can be used to assess the performance of the long-term care system in Korea, and the framework and its methodological approach can be benchmarks for other countries developing their own framework.

**Conclusions:**

It is critical to reconcile and prioritise various stakeholders’ views and information needs as well as to balance methodological rigor with practical usefulness and feasibility in the development and implementation of a long-term care performance monitoring system.

## Introduction

### Background

South Korea is a country with one of the most rapidly aging populations in the world. People aged 65 or older compose 11% of the whole population, but the proportion is expected to increase up to approximately 40% by 2050 [1]. Traditionally, in Korea, an Asian country that values filial piety, caring for older family members is considered the most important family responsibility and is mainly done by the first-born son and his wife [2]. However, this social norm has drastically changed following a rapid industrialisation and urbanisation as well as the experienced societal changes in family structure and an increase in women’s labour participation [3].

Responding to the increasing social responsibility for caregiving to the older population, a public long-term care (LTC) system financed by a social long-term care insurance (LTCI) scheme was introduced in 2008. About 7.5% of people aged 65 or older received LTC under the LTCI in 2016 [4]. Since the inception of the LTCI, policy has focused on building an infrastructure for the public LTC system nationwide and recently put effort into expanding coverage for people with dementia and strengthening quality monitoring [5]. As yet, little work has been done on assessing the system-level performance of LTC under the public LTCI, which could facilitate policy-makers’ monitoring of the LTC system objectively and consistently, identify current issues and future challenges, and informing evidence-based policy-making in Korea. Developing a performance framework including internationally relevant indicators could also allow the monitoring of its performance in a comparative way with other countries that have similar LTC systems; this could facilitate cross-national policy learning and evaluation.

LTC system performance assessment (LTCSPA) has often been considered to be the same as LTC quality evaluation, yet this is not actually the case. The latter is commonly an evaluation performed at the LTC provider (institution) level, whereas the former tends to be an evaluation performed at the national and regional levels of an LTC system. The two assessments may differ in many ways, including their goals and units of assessment as well as the stakeholders and audience of the evaluations. There are a few existing frameworks for LTCSPA in the United States, Canada, Europe, Japan and South Korea, some of which were proposals only while others were implemented [6–11]. The frameworks have shared goals to better understand their LTC system(s) and to inform various stakeholders of their performance. However, each is unique because the policy contexts and characteristics of each LTC system to be assessed are not the same; this is well reflected in variations in the dimensions and performance indicators across the frameworks.

This study aimed to report the process and results of a performance assessment framework for the LTC system in Korea, including theoretical consideration, review of domestic and international studies, and expert input, followed by a feasibility test. Lessons from the development process and recommendations for the future implementation process will be discussed with consideration to the relevance of Korea’s experience for other countries with similar policy needs.

### Performance assessment of long-term care systems

The field of health system performance assessment (HSPA) research has been established since the publication of the World Health Report by the WHO and is quite active in the United States and in European countries as well as internationally through the OECD Health Care Quality Indicator (HCQI) project [12–15]. LTCSPA is an emerging field compared to HSPA, yet the ultimate goals of conducting HSPA or LTCSPA are not likely to be very different. The purpose of LTCSPA is to support information-based communication among diverse stakeholders and the establishment of responsible relationships related to the system’s performance. It is challenging and somewhat infeasible to design an LTCSPA information system that satisfies all the information needs of the diverse stakeholders [16]. However, a framework and a concise set of indicators for LTCSPA that are theoretically/conceptually valid and relevant can provide a bird’s eye view on the current status of a complex LTC system and guide policy efforts to develop the system. The goals of the LTC system to be assessed should first be identified, and the performance dimensions and indicators in the performance assessment framework to be developed should be aligned with the performance goals [16]. It would be ideal to develop a set of indicators that can assess the structure, process and outcome aspects of a system [17]. In addition, it is important to consider the balance of indicators allowing for cross-national comparison and those reflecting country-specific measurement needs and context.

The literature further recommends some key considerations regarding the development of a performance assessment framework and indicators [18]. First, the framework and indicator sets should have policy relevance and usefulness to the stakeholders. The indicators reflect the context and issues of the systems to be assessed; therefore, the indicators should measure aspects and phenomena that are meaningful nationally and regionally. Second, the indicators are to be theoretically well established and analytically sound. The validity of the measures should meet international standards, and the indicators should be easy to understand and interpret. Third, the indicators must have measurability, which means that data to produce the indicators should be available and able to be updated readily in a timely manner. The quality of the data needs to be ensured, for which a reliable process of data development and regular updates are to be in place. In addition, the literature also suggests constructing a set of performance indicators balancing macro-, meso- and micro-level data [18]. These are useful guides for developing performance frameworks and indicators for LTCSPA, but it is hard to meet all these recommendations in practice [16].

### The public long-term care insurance in Korea

The public LTCI programme in Korea is governed by the Ministry of Health and Welfare (MOHW) and the National Health Insurance Service (NHIS) in collaboration with local governments. The MOHW makes the overall plans for financing and provision of the programme, and the NHIS has the actual responsibility of administering the programme. The LTCI is financed by contributions (60–65%), government subsidies (about 20%) and co-payments by service users (20% for institutional services and 15% for home-based services). The level of contributions was determined with regard to the set rate of the health insurance premium (7.38% in 2018). Public spending on LTC (health and social components) was about 0.8% in 2014, which is lower than the OECD average (1.4%) and also that of other countries with social LTCI (e.g. 4.3% in the Netherlands, 2.1% in Japan and 1.1% in Germany) [19].

The public LTCI programme targets people aged 65 and over and those under 65 with senile diseases who have a certain level of dependency regardless of income level or the availability of family care. Care needs are assessed through a nation-wide, standardised care needs assessment system including a 52-item eligibility test covering physical, cognitive, behaviour, nursing care and rehabilitation; the LTCI programme currently runs a six-level eligibility system (Level 1 refers to the highest needs) [20]. The population coverage of the LTCI programme was about 2.9% of people aged 65 or older at the inception of the programme in July 2008, and the coverage had more than doubled, up to 7.5% of older people, by December 2016 [4]. The coverage rate is still regarded as low compared to Japan and Germany, whose public LTCI programmes covered about 18% (in 2017) and 12% (2.6% in institutions, 9.5% at home in 2016), respectively, of the older adult population [19, 21].

Unlike Japan and Germany, a nation-wide quality monitoring system for the public LTCI programme in Korea was immediately introduced in 2009, which was possible because the Korean LTCI programme is a centralised programme operated by the NHIS, a single public insurer that also runs the national health insurance programme, which has a strong quality monitoring and assurance scheme. Quality monitoring is conducted in five domains – management of institutions, environment and safety, rights and responsibilities, process of services, and outcome of services. A major caveat is the lack of process and outcome indicators to assess quality of care at the person level [22]. Consistent efforts to improve the quality of the quality monitoring system have been made by the NHIS.

## Methods

We developed an LTC system performance framework including performance goals, dimensions and indicators for the public LTCI programme in Korea through a six-step process, as follows, based on the approaches of existing studies [6, 8, 16, 23, 24].

Step 1: We set the goals and scope of the performance assessment by examining the current policy goals of the public LTC system and stakeholder and expert input as well as reviews of the relevant documents.

Step 2: We systematically reviewed existing domestic and international frameworks for LTCSPA – theoretical and conceptual foundations/rationales, the goals and scope of the assessments, organisation of the frameworks, criteria for indicator selections, the process of indicator development, etc.

Step 3: We identified the goal and domains of the current LTCSPA framework in Korea through document analysis of the LTCI Act, national long-term development plans and key statistics of the LTCI as well as expert input from researchers, policy-makers and administrators. We also reviewed a wide range of literature, including texts on the laws and administrative orders, policy reports and published/grey papers on the system. For each domain, a set of preliminary indicators was developed through multiple iterations of review and selection of indicators discussed in the existing domestic and international LTCSPA literature. We also reviewed some indicators for LTC systems in HSPA frameworks (e.g. the OECD Health Care Quality Framework), as an LTC system is often regarded as part of the health system in a country [25, 26]. We considered other indicators, too, that were originally for health systems but were still relevant for LTC systems either as they were or at least conceptually (e.g. ratio of public expenditure, health-related quality of life), and we adopted and/or modified these indicators for our purposes.

Step 4: In order to obtain face validity, a two-round Delphi survey was given to approximately 20 stakeholders of the Korean LTCI, including 15 academics in various fields (medicine, nursing, public health, social work and others) and also policy-makers and administrators from the Ministry of Health and Welfare and the NHIS. The results of the Delphi survey were further closely reviewed, and issues and concerns raised in the survey were discussed through face-to-face meetings with experts on Korean LTC systems and policy and performance measurement research experts with diverse backgrounds in terms of discipline, affiliated institution and role.

Step 5: A pilot assessment was conducted to examine the feasibility of generating data for the set of performance indicators selected through Step 4. We analysed administrative data routinely collected and managed by the NHIS, including care needs assessment data and utilisation data, and de-identified beneficiary profile data from the LTCI and the National Health Insurance (NHI). Primary data collection was conducted for four indicators; we developed, pilot-tested and revised a survey form including existing instruments. We applied the survey tool to examine its feasibility for collecting such data and received feedback from 54 survey participants, LTCI beneficiaries, families and practitioners who received or provided services under LTCI at home or in LTC facilities.

Step 6: A meeting with diverse stakeholders was organised to gather their opinions, including the research team, which comprised university professors of health and LTC policy, social welfare, statistics and others; representatives from MOHW, the National Committee of Long-term Care, the insurer (the Health Insurance Policy Research Institute and the administrative departments under NHIS), a beneficiary (consumer) advocate group (Green Consumer Network in Korea), provider organisations (Korean Convalescent Hospital Association, Korea Federation of Senior Welfare, and the Korea Association of Long Term Care Center for Senior Citizens), and academics (university professors of health and LTC policy). In the meeting, the research team presented an overview of the study including the aim and concept of LTCSPA, and the final framework and set of performance indicators for LTCSPA was proposed. A questions and answers and discussion sessions followed. This study was approved by the institutional review board at the institution where the first author was affiliated.

## Results

### The proposed framework of LTC system performance assessment: goals, domains and indicators

The proposed framework for performance assessment of the LTC system under the public LTCI in Korea consists of a goal, four domains and 28 indicators (Table 1). The performance goal of the LTC system under the public LTCI is to improve the health and quality of life of the beneficiaries and their families through good coverage and quality services, as well as the sustainability of the system. The four performance domains of the proposed framework are coverage, quality, health and quality of life, and system sustainability (Table 1). First, the coverage domain assesses the extent of coverage and cost sharing by beneficiaries, for which eight performance indicators (C1–C8) were developed. The two subdomains of the coverage domain are ‘coverage rate of LTCI’ and ‘financial burden’. The former is basically to measure the extent of population and service coverage by the public LTCI, and the latter aims to measure how much financial burden is taken on by the public LTCI, which in turn ensures improvements in affordability of LTC services for the elderly and their families (e.g. decreasing co-payments and out-of-pocket costs).

**Table 1:** The goal, domains and subdomains of the Long-Term Care System Performance Assessment (LTCSPA) framework for the public Long-Term Care Insurance (LTCI) system in Korea

Performance goal	The public long-term care (LTC) system should be sustainable and improve the health and quality of life of the beneficiaries and their families through good coverage and quality services			
Domain	○ Coverage	○ Service quality	○ Health and quality of life	○ Sustainability
	- To assure good coverage of population and services while reducing the financial burden on beneficiaries	- To provide quality care and a good user experience and promote coordinated care that promotes aging in place	- To maintain and improve the health and quality of life of beneficiaries and their families	- To assure the sustainability of the LTC system through financial management and efficient operation with support from the public
Subdomain	○ LTCI coverage rate	○ Quality of care	○ Health quality of life	○ Financial management
	○ Financial burden	○ Community-centred care		○ System efficiency
		coordination and integration		○ Public acceptance

‘Quality’ is the second domain of the framework, as the provision of high quality LTC is also a key policy goal of the first and second Basic Plans for the LTCI in Korea [27, 28]. It consists of two subdomains: one is ‘quality of care’ and the other is ‘community-based care coordination and integration’ (hereafter ‘care coordination’). The quality of care sub-domain is measured by a set of three indicators related to staffing ratios (total formal LTC workers, nurses, and personal carers; Q1–Q3) and also a user experience indicator (Q4) to assess the responsiveness of the LTCI system. Care coordination, meanwhile, reflects the fact that the LTCI law prioritises home and community-based care over institution-based care and also prioritises integrated LTC-medical care to prevent the deterioration of the health status of LTC users (Q5–Q9).

‘Health and quality of life’ is the third domain, as these are the ultimate outcomes to achieve with LTCI (Table 2). Article 1 of the LTCI law identifies health as the primary goal of the programme and states improvement in the quality of life of service users and their families is the ultimate purpose of the implementation of the LTCI [29]. As for the ‘health’ sub-domain, two performance measures were proposed – maintenance and improvement of care-need level (H1) and hospitalisations (possibly due to aggravation of conditions; H2). System performance for the ‘quality of life’ subdomain is assessed using EQ5-D (H3), an internationally comparable patient-reported outcome measure (PROM) tool, and also by caregiving time by the family (H4) and the proportion of family caregivers who report a reduced burden of caregiving (H5); these measures aim to assess changes in the objective and subjective burden of family caregiving, respectively.

**Table 2:** The final set of performance indicators for the proposed Long-Term Care System Performance Assessment (LTCSPA) framework

Domain	Subdomain	No.	Indicator	Type of data source	Use of indicators			
					Level		Distribution by	
					Core	Int’l Comp.	Income	region
Coverage	LTCI coverage rate	C1	Certified rate: Eligible beneficiaries as a share of people aged 65 and over	LTCI statistics yearbook	√	√		√
		C2	Utilisation rate: LTCI recipients as a share of all eligible beneficiaries	LTCI statistics yearbook				√
		C3	Utilisation rate: LTCI recipients as a share of people aged 65 and over with activities of daily living limitations	Other data				
		C4	HCBS users as a share of all beneficiaries	LTCI statistics yearbook				√
		C5	Institutional care service users as a share of all beneficiaries	LTCI statistics Yearbook				√
	Financial burden	C6	Public spending as share of total LTCI expenditure	LTCI statistics yearbook	√	√		
		C7	Annual LTCI expenditure per capita	LTCI statistics yearbook		√		
		C8	Out-of-pocket LTC spending as a share of final household consumption	Primary survey				
Quality	Quality of care	Q1	Total formal LTC workers per institutional care user	LTCI statistics yearbook	√			√
		Q2	Nurses per institutional care user	LTCI statistics yearbook		√		√
		Q3	Personal carers per institutional care user	LTCI statistics yearbook				√
		Q4	User experience	Primary survey			√	
	Community-centred care coordination and integration	Q5	HCBS users as a share of all users	LTCI micro administrative data	√	√	√	√
		Q6	Multiple HCBS users as a share of all HCBS users	LTCI micro administrative data		√	√	
		Q7	Institutional care users with less-severe level (care-need level 3 and lower) as a share of all institutional care users	LTCI micro administrative data			√	√
		Q8	Admission to institutions among HCBS users	LTCI micro administrative data		√		√
		Q9	Admission to hospitals among HCBS users	LTCI micro administrative data			√	√
Health and quality of life	Health	H1	Proportion of LTCI users whose care-need level was improved or maintained	LTCI micro administrative data	√	√	√	√
		H2	Hospital admissions of LTCI users	LTCI micro administrative data			√	√
	Quality of life	H3	Health-related quality of life	Primary survey	√	√	√	√
		H4	Average caregiving times for family caregivers of HCBS users	Primary survey		√		
		H5	Proportion of family caregivers who reduced their burden of caregiving	Primary survey				
Sustainability	Financial management	S1	LTCI expenditure as a share of GDP	LTCI statistics yearbook	√	√		
		S2	Ratio of surplus to income of the public LTCI	LTCI statistics yearbook				
	System efficiency	S3	LTCI expenditure per capita on the maintenance or improvement of care-need level	LTCI micro administrative data	√		√	√
		S4	Ratio of health insurance expenditure per capita by LTCI users compared to non-users	LTCI micro administrative data		√	√	√
	Public acceptance	S5	Perceptions of the LTCI programme by the public	Other data	√			
		S6	Public’s willingness to use LTCI services	Other data				

*HCBS* Home- and community-based care service, *LTC* long-term care, *LTCI* Long-Term Care Insurance

The final performance domain, ‘sustainability’, includes three subdomains. The ‘financial management’ subdomain measures LTCI expenditures as a share of GDP (S1) and the ratio of surplus-to-income of the public LTCI (S2). The ‘system efficiency’ subdomain aims to measure the cost (input)–quality (output) relationship, assessed by two performance indicators, LTCI expenditure per capita on the maintenance or improvement of care-need level (S3) and ratio of health insurance expenditure per capita by LTCI users compared to non-users (S4). The last subdomain is ‘public acceptance’, a kind of proxy for the system’s responsiveness, measured by two indicators, the public’s perceptions of the LTCI programme (S5) and the public’s willingness to use LTCI services (S6). Rather than focusing on users’ experience, the indicators aim to assess the overall acceptance of the LTCI by all subscribers, that is, by the public, as the LTCI is a mandatory social insurance in Korea.

### Data sources to generate the indicators

To generate relevant statistical values for these indictors, four categories of data sources were used, namely existing national statistics, micro LTCI and NHI claims data, primary user survey data, and others (Table 2). The data needed to calculate many of the proposed indicators (11 out of 28) can be easily extracted from the LTCI Statistics Yearbooks that are published by the NHIS every year and are open to the public. Most of these indicators are under the coverage and sustainability domains, such as certified rate (C1) or ratio of total expenditure-to-GDP (S1). Second, nine indicators can be generated from LTCI/NHI administrative data, including the ratio of multiple home- and community-based care service (HCBS) users (Q6) and rate of acute hospitalisation (H2). This is possible because both the LTCI and the NHI are operated by the NHIS, which also maintains lifelong health and LTC administrative records on all Koreans [5]. Third, all three performance indicators (H3, H4 & H5) in the ‘quality of life’ subdomain and the user experience indicator (Q4) in the ‘quality of care’ subdomain requires a primary survey or the use of existing survey data (the national survey on elderly life conditions) [30]. We confirmed that it was feasible to generate all the statistical values for the indicators from the various data sources proposed above in the pilot study.

### Use of performance indicators

**Fig. 1:**
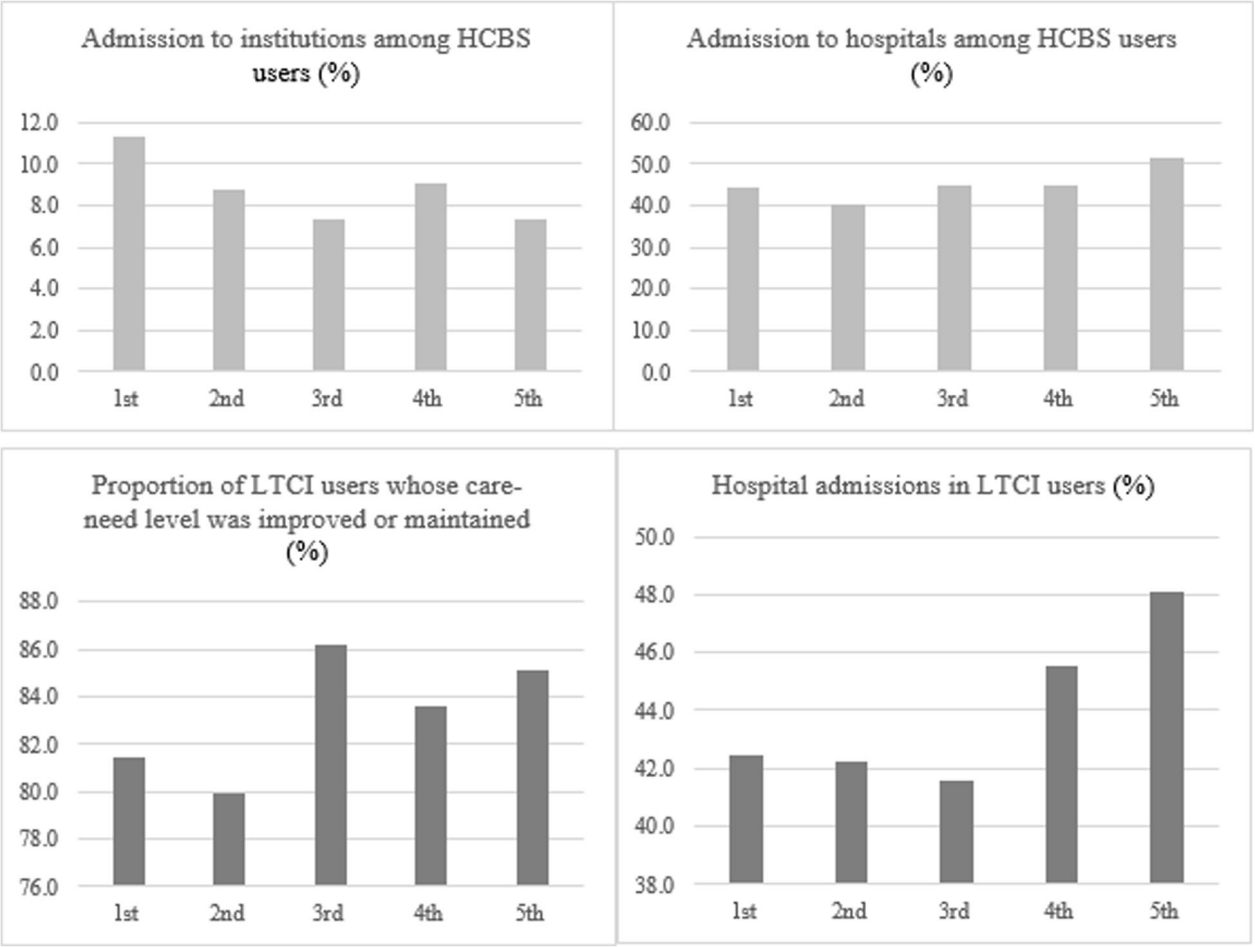
Examples of variations in long-term care (LTC) system performance by income level from a pilot assessment. Note: The x-axis is income-level quartile, from the richest (1st quartile) to the poorest (5th) based on insurance premium; the y-axis is the percentage (%) of relevant users

The experts and stakeholders asked the research team to consider ways to improve the usability of the proposed indicators (Table 1). To meet this request, we proposed three subsets of indicators labelled as core, international comparison and equality indicators, respectively, based on existing evidence and experts’ input. The first subset involves 10 core indicators across all four domains that can be used for wider communication with the general public or policy-makers [18]. The second subset of 12 indicators is for international comparison; these indicators are comparable with the Japanese LTC assessment system, Germany’s minimum staffing standards for nursing homes, or OECD health data [31]. The third group is to assess the equality dimension of LTCI programme performance. Income and region are the two selected strata for examining performance variations in the LTCI programme, for which 10 and 16 indicators were proposed, respectively. Figure 1 is an example of analysis results from the pilot feasibility study, which shows how the provision/use of LTCI programmes measured by the rate of admission to institutions among HCBS users (Q8) and the rate of admission to hospitals among HCBS users (Q9) vary by income level measured using insurance-premium level as a proxy.

## Discussion

We have reported the process and results of developing a framework to assess the performance of the LTC system financed by the public LTCI with universal coverage for older people in Korea. The performance goals for the assessment framework were identified based on a review of the LTCI laws and policy documents regarding LTCI in Korea and consultations with experts; the goals guided the development of the performance assessment framework and were received well and did not change throughout the meetings with experts and stakeholders in the study.

The four domains of the performance framework have some similarities and differences with existing frameworks for LTCSPA. The coverage domain was selected because coverage expansion was a policy priority in the second national plan for the Korean LTCI [28] and also is a shared key policy agenda for OECD countries; the indicators for level of LTC coverage (C4 and C5) in our framework are also performance indicators adopted by the OECD [19]. System sustainability is also an important performance domain of the LTCI in Japan [11].

On the other hand, the ‘effective transitions’ domain in the Long-Term Services and Support scorecard proposed by the AARP foundation in the United States was not a separate domain in our framework [6]; this is due to different policy priorities in the two countries. We instead include an indicator on admission to hospitals among HCBS users (Q9) in the care coordination domain. Similarly, ’choice of providers and service settings/type (e.g. cash or in-kind)’ is a major performance domain assessed in the United States and Europe, where often the services are financed by taxes by local authorities or by Medicaid (United States); rationing of care, including prioritising people eligible for the limited institutional LTC, is a key policy agenda, which often results in a long wait-time for older people depending on the family’s caregiving burden. In contrast, South Korea’s social LTCI provides relatively easy access and free choice of provider and services to all entitled older citizens and their family members, which could result in misuse and overuse. Thus, choice in Korea is less likely to be an urgent system performance issue at present, while the narrow coverage (deciding who is in or not) is more so; therefore, population coverage became a key domain of the performance framework developed.

As for the performance indicators, the 10 core indicators can be a concise set for wider communication with policy-makers and the public to show a snapshot of the performance of the LTC system under the LTCI. The core indicators consist of key indicators from the four domains, most of which can be produced from routinely collected LTC administrative data. Domestically, the total set of indicators, including country-specific indicators, can be used to develop the national LTC plan and guide the revision of policies every 5 years based on Article 6 of the LTCI Act [29]. These indicators can be used to produce web-based public reports similar to the Nursing Home Compare website by the United States Centers for Medicare and Medicaid Services and the Your Health System website by the Canadian Institute for Health Information [32, 33].

The 12 international indicators can be used for cross-national comparison of LTC performance among OECD countries (in particular, those countries with similar social insurance-based LTC systems), considering that the current LTC-related indicators under the OECD HCQI project are limited. Comparisons of LTC systems using these indicators may not be straightforward; different historical paths, levels of system development, policies and political contexts should be also considered in the interpretation of findings. Lastly, the equity aspects of LTC system performance are an important policy agenda that has rarely been addressed in the existing literature in Korea. The 10 and 16 indicators for variations in LTC system performance based on income and region can be used for such a knowledge gap. The United States State Scorecard on Long-Term Services and Support reported disparities between states, and the European ANCIEN project also included ‘equity in resource allocation’ as a performance domain [6, 8].

### Strengths and limitations

This study has strengths and limitations. The domains and indicators of the proposed framework may not be relevant permanently, and periodic review and updates are needed. Most indicators in our performance framework can be generated from routinely collected LTCI administrative data. This approach has advantages in cost and effort of data collection, but the quality of administrative data needs to be monitored. The performance indicators in the health and quality of life domain should be strengthened through collection of person-level care process and health outcomes data. The usability as well as challenges in implementation of LTC performance monitoring and public reporting systems should be further tested, based on which refinement of the framework is recommended.

## Conclusions

This study developed an LTCSPA framework and indicators through theoretical review, review of existing frameworks from other countries, and input from expert groups as well as feasibility tests in Korea. The methodological approaches and the proposed framework can be a foundational work for establishing an evidence-based performance monitoring system for Korea’s public LTCI, which reached its 10th anniversary in 2018. Based on the research findings, lessons can be drawn for countries with similar policy needs for assessing the performance of an LTC system. First, methodologically, securing quality data to produce performance indicators is critical. South Korea is one of the OECD countries with the most advanced health information systems [34], so we took advantage of the health and LTC big data that the NHIS has maintained and the existing nationwide social and health survey data. Countries should assess their existing data infrastructure and plan ahead to build such a foundation for evidence-based policy-making through LTCSPA. In spite of the relatively good data infrastructure in Korea, it was still quite challenging to put all the data together from various sources and generate the performance indicators required in the study. Having a master plan and building a tailored information system for LTCSPA from the beginning is recommended.

Second, countries should clarify the policy needs and goals of their own LTCSPA first, based on which the performance assessment framework needs to be developed. In addition, for countries with limited resources, it may be wise to begin by assessing the financial and sustainability domains of LTC system performance, and then extend to other domains. Person-level functional assessment data are valuable for assessing system performance in the health and quality of life as well as quality of care domains, but collecting such data requires significant investment of financial and human resources. The United States and Canada are good examples for establishing standardised health and functional assessment databases for LTC users across the nation and/or province(s) [35, 36]. Using these databases, quality monitoring and public reporting are actively done in the United States and Canada for various stakeholders, although there are also concerns about the quality of data and the usefulness of such surveillance systems [37]. It is also critical to provide education, training and/or support programmes for data assessors.

Third, it is necessary to balance scientific rigor and administrative usability as well as to reconcile different stakeholders’ views and priorities for system performance in determining the breadth and depth of the domains and performance indicators. Making a perfect agreement among various stakeholders is unrealistic, but this study shows that reaching a reasonable consensus through the multiple steps we took is possible; such an approach can be a benchmark for other countries. In addition, developing a governance body and process to review and update the framework and indicators depending on policy needs as well as data availability is recommended. Lastly, successful implementation of an LTC performance monitoring system would likely require vision, policy will and careful planning with practical considerations.

## Data Availability

The datasets used and/or analysed during the current study are available from the corresponding author on reasonable request.

## Abbreviations

HCBS: Home- and community-based care service
HCQI: Health Care Quality Indicator
HSPA: Health System Performance Assessment
LTC: Long-Term Care
LTCI: Long-Term Care Insurance
LTCSPA: Long-Term Care System Performance Assessment
MOHW: Ministry of Health and Welfare
NHI: National Health Insurance
NHIS: National Health Insurance Service
